# Hydrogen Sulfide, an Endogenous Stimulator of Mitochondrial Function in Cancer Cells

**DOI:** 10.3390/cells10020220

**Published:** 2021-01-22

**Authors:** Csaba Szabo

**Affiliations:** Chair of Pharmacology, Section of Medicine, University of Fribourg, CH-1700 Fribourg, Switzerland; csaba.szabo@unifr.ch

**Keywords:** bioenergetics, ATP, gasotransmitters, DNA repair

## Abstract

Hydrogen sulfide (H_2_S) has a long history as toxic gas and environmental hazard; inhibition of cytochrome c oxidase (mitochondrial Complex IV) is viewed as a primary mode of its cytotoxic action. However, studies conducted over the last two decades unveiled multiple biological regulatory roles of H_2_S as an endogenously produced mammalian gaseous transmitter. Cystathionine γ-lyase (CSE), cystathionine β-synthase (CBS) and 3-mercaptopyruvate sulfurtransferase (3-MST) are currently viewed as the principal mammalian H_2_S-generating enzymes. In contrast to its inhibitory (toxicological) mitochondrial effects, at lower (physiological) concentrations, H_2_S serves as a stimulator of electron transport in mammalian mitochondria, by acting as an electron donor—with sulfide:quinone oxidoreductase (SQR) being the immediate electron acceptor. The mitochondrial roles of H_2_S are significant in various cancer cells, many of which exhibit high expression and partial mitochondrial localization of various H_2_S producing enzymes. In addition to the stimulation of mitochondrial ATP production, the roles of endogenous H_2_S in cancer cells include the maintenance of mitochondrial organization (protection against mitochondrial fission) and the maintenance of mitochondrial DNA repair (via the stimulation of the assembly of mitochondrial DNA repair complexes). The current article overviews the state-of-the-art knowledge regarding the mitochondrial functions of endogenously produced H_2_S in cancer cells.

## 1. Hydrogen sulfide (H_2_S), an Endogenous Mammalian Biological Mediator

Over the majority of the last century hydrogen sulfide (H_2_S) has been viewed as an environmental toxin and biological hazard. H_2_S (administered as an inhaled gas, or systemically, in aqueous solutions) exerts various toxicological effects in all mammalian organisms including humans. An extensive body of literature exists which focuses on the toxicological aspects of H_2_S [1,2,3,4,5]. Relevant for the current article is the fact that the molecular mode of H_2_S’ toxic action is largely (although not exclusively) attributed to its ability to inhibit mitochondrial Complex IV (cytochrome c oxidase), which, in turn, shuts down mitochondrial electron transport and inhibits aerobic ATP generation. The (largely reversible) binding of H_2_S to the cytochrome a3 prosthetic group of Complex IV has been extensively characterized [6,7]. Although Complex IV inhibition by H_2_S is usually viewed in the environmental toxicological context, there are some pathophysiological conditions—for example Down syndrome, where increased levels of endogenously produced H_2_S can inhibit Complex IV [8,9].

Over the last two decades, the field of H_2_S has undergone a significant transition, whereby various endogenous H_2_S-producing enzymes have been recognized and characterized, and a broad spectrum of biological roles of H_2_S has been identified. The timeline of H_2_S research and the emergence of H_2_S as an endogenous mammalian mediator has been covered in specialized review articles [10,11]. Moreover, the diverse biological roles of H_2_S in mammals in the regulation of the cardiovascular, nervous and immune system, and the biochemistry and pharmacology of various H_2_S-producing enzymes—a stand-alone field that consists of over 10,000 published papers—is covered in specialized review articles [12,13,14,15,16,17,18,19,20,21,22,23,24,25,26,27,28,29,30]. Briefly, H_2_S in mammalian cells and tissues is produced by three principal enzymes: cystathionine-β-synthase (CBS), cystathionine γ-lyase (CSE) and 3-mercaptopyruvate sulfurtransferase (3-MST), with several additional enzymes (as well as non-enzymatic reactions and H_2_S-producing bacteria in the bacterial microbiota) also contributing. CBS, CSE and (indirectly) 3-MST all utilize sulfur-containing amino acids as their enzymatic substrates; the biochemistry and the molecular regulation of each enzyme in health and disease has been extensively reviewed in specialized articles [31,32,33,34,35]. H_2_S is a diffusible mediator that reaches various cellular compartments, and can also exit the cell that produces it. Thus, it can exert biological actions in autocrine as well as paracrine manners. H_2_S can also react with other labile, diffusible small molecules (such as nitric oxide, peroxynitrite, superoxide, hydrogen peroxide and others) to produce various secondary and tertiary reactive species: thus, H_2_S is now viewed as a component of the “reactive species interactome” [36,37]. While we cannot attempt to outline the complex roles and regulation of H_2_S and the H_2_S-producing enzymes in the current article, a brief discussion of the following aspects will be useful in the context of the current, focused review article:

There are important parallels between bacterial sulfur biochemistry and the mitochondrial roles of H_2_S. This is not at all surprising considering that mitochondria, from an evolutionary standpoint, are, in fact, modified bacteria. Bacteria (similar to present-day mitochondria) are known to produce and/or utilize H_2_S; these processes involve several evolutionarily conserved enzymes [10,38].

The mammalian H_2_S-producing enzymes are (partially) mitochondrial. While 3-MST is often referred to as a “mitochondrial enzyme”, it actually has both mitochondrial and cytosolic localization; the other two major enzymes can either have low-level mitochondrial baseline localization, and/or can translocate to mitochondria under various specialized (pathophysiological) conditions (including cancer) [39,40,41]. However, mitochondrial localization of a H_2_S-producing enzyme is not an absolute requirement for mitochondrial effects of H_2_S, since this mediator can readily diffuse from one intracellular compartment to another [28]. 

For the sake of simplicity, one tends to simply refer to “H_2_S”, when discussing the complex biochemical products of CBS, CSE and 3-MST. However, the reality is that in a cellular environment, due to a complex series of reactions, multiple reactive sulfur species exist, as well as various hybrid (“S/N”) species. These diverse species can have different chemical reactivity and diverse (but often overlapping) sets of molecular targets. Polysulfides (small segments of S-Sn chains) represent a special group of reactive sulfur species; while CBS and CSE is primarily viewed as “H_2_S synthases”, 3-MST’s principal product is polysulfide (although, each enzyme generates a mixture of these species in the intracellular environment) [28,34,42].

While H_2_S (and polysulfides) have multiple intra- and extra-cellular molecular targets, one of the principal modes of reactive sulfur species’ action is sulfhydration (a posttranslational modification of protein cysteines), which is primarily catalyzed by polysulfide (rather than H_2_S) [34,42,43].

“Protein sulfhydromes” (collections of sulfhydrated proteins) have recently been characterized in various cells and tissues; thousands of proteins are subject to this modification [44,45,46,47]. Some of the better-characterized enzymes that are known to be functionally affected by sulfhydration include K_ATP_ channels (regulating vascular tone, angiogenesis and many other processes), nuclear factor kB (regulating signal transduction), and Keap 1 (regulating NRF2 activation and thus inducing a generalized cellular antioxidant response, responsible for cytoprotection, preconditioning and other responses) [43,44,45,46,47]. 

It should be mentioned that a significant reprogramming of the cellular sulfur metabolism (which includes alterations in cysteine catabolism and metabolism, cysteine transport, methionine homeostasis, and many other aspects) occurs in cancer cells [48,49]; the mitochondrial roles of H_2_S in cancer (reviewed in the current article), therefore, should be viewed in this broader context.

Generally, H_2_S is known to have a bell-shaped (or biphasic) biological character, with low-to-mid levels exerting one type of biological response (in many cases, regulatory, protective or stimulatory), while higher (toxicological) levels of H_2_S often exerting the opposite effects (which are, in many cases, related to the inhibition of mitochondrial Complex IV, as discussed above) [28].

Finally—as extensively discussed previously [24,28,31,41]—it should be emphasized that many of the experimental findings discussed in the following sections rely on pharmacological inhibitors of CBS (such as aminooxyacetic acid, AOAA) and of 3-MST (such as 2-[(4-hydroxy-6-methylpyrimidin-2-yl)sulfanyl]-1-(naphthalen-1-yl)ethan-1-one, HMPSNE). These agents have low micromolar potency on their respective enzymatic targets in biochemical assays in vitro, and have acceptable cell uptake. (When cells are incubated with these agents in mid-to-high micromolar concentrations, cellular H_2_S production is significantly suppressed). However, these compounds can also inhibit other enzymes (for instance, AOAA also inhibits CSE, and indirectly, 3-MST), and their selectivity and specificity, as well as their bioavailability and metabolism are incompletely understood. It is, therefore, always preferable to test the potential reversibility of the biological effects of these inhibitors with H_2_S donors, and to complement the pharmacological studies with genetic studies (e.g., CBS, CSE or 3-MST silencing). 

## 2. Upregulation of Various H_2_S-Producing Enzymes in Cancer Cells 

In 2013, we have discovered that CBS is upregulated and H_2_S generation is increased in primary human colon cancer tissues compared to the surrounding (nominally healthy) tissues [50]. Similarly, we have noticed that several human colon cancer cell lines have highly expressed CBS levels [50]. Over the subsequent seven years, these findings have been confirmed and extended to many different tumor types: it is now clear that CBS, and/or CSE and/or 3-MST is overexpressed in many forms of cancer. Table 1 shows an overview of the currently published body of literature in this respect [40,41,51,52].

What, then, is the functional relevance of this increased H_2_S production in cancer cells? The functional role of CBS- or 3-MST-derived H_2_S in colon cancer cells has been studied extensively. Utilizing knockdown of various H_2_S-producing enzymes and/or pharmacological inhibitors, and/or forced overexpression of H_2_S-producing enzymes into non-transformed cells, our group as well as several other groups of independent investigators have demonstrated that cancer cells upregulate their H_2_S-producing capacity to help their bioenergetic function (glycolysis as well as oxidative phosphorylation, see below), to maximize ATP generation in support of their increased (uncontrolled) growth, proliferation and migration [50,51,52,53,54,55,56,57,58,59,60,61,62,63,64,65,66,67,68,69,70,71]. 

Moreover, endogenously produced H_2_S in colon cancer cells stimulates various cellular signalling pathways, reinforces stemness, provides protection against anticancer chemotherapeutic agents, and helps maintaining cancer cells in a mesenchymal (as opposed to epithelial) state, in support of cellular mobility, invasion and metastasis. H_2_S is an endogenous vasodilator [72,73,74] and pro-angiogenic factor [75,76,77,78]; tumor-derived H_2_S, therefore, also exerts local (paracrine) actions in the tumor microenvironment, which are important to increase blood flow and nutrient supply to the growing tumor tissue [39,40,50]. The above-listed roles of H_2_S are not restricted to colon cancer: similar roles of H_2_S have also been demonstrated in a variety of other cancer types [40,51,79,80,81,82,83]. Some of the enzymes that are known to be regulated by H_2_S, and which, in turn, serve the increased metabolic demands of the tumor cell are listed in Table 2 [19,60,66,71,84,85,86,87,88,89,90,91,92,93,94,95,96,97,98,99,100,101].

All of the above actions of H_2_S can also be viewed, in a broader context, as one of the multitude of mechanisms that cancer cells mobilize, in order to serve their extreme bioenergetic demand. According to the original version of the “Warburg hypothesis”, cancer cells switch to glycolysis from oxidative phosphorylation. However, more recent data indicate that many cancer cells, while upregulating glycolysis (as well as glutaminolysis, and many additional metabolic pathways) can also maintain or even increase their aerobic ATP generation via the stimulation of mitochondrial electron transport [101,102,103,104,105,106]. To anthropomorphize: maximizing ATP generation is the cancer cell’s primary “metabolic goal”: whatever biochemical mechanism serves this goal—even if it is ”wasteful” or perhaps useful in the short-term but detrimental in the longer-term—will be deemed ‘good enough’ to satisfy the “short-term thinking” of the cancer cell.

## 3. H_2_S, a Mitochondrial Electron Donor and Stimulatory Bioenergetic Factor in Cancer Cells

Mitochondrial respiration is responsible for the majority of ATP generation in eukaryotes. Electron transport along mitochondrial electron transport chain complexes I, II, III and IV creates an electrochemical proton gradient, which acts as the driving force for ATP generation; the proton gradient is “harvested” by ATP synthase (mitochondrial Complex V). Pioneering work of Bouillaud and colleagues, starting in 2007, demonstrated that H_2_S can serve as an alternative mitochondrial electron donor; in fact, H_2_S was characterized as the ”first inorganic substrate for human cells” [88]. The initial studies have been conducted in nominally normal cells (i.e., non-transformed intestinal epithelial cells), and was discussed in a physiological context (i.e., the function of the gut epithelial cells to protect against the H_2_S generated by the intestinal microbiome, and to utilize it as their own bioenergetic “fuel”) [89,107,108,109,110]. However, further studies demonstrated that the same basic biochemical mechanisms are also operative in cancer cells; in this case the H_2_S that drives the mitochondrial electron transport is not the consequence of external (i.e., bacterial) sources but is produced internally (through the upregulation of the various H_2_S-producing enzymes; Table 1). For instance, in colon cancer cells CBS silencing suppresses basal mitochondrial function (oxygen consumption, ATP generation) [50] and similar effects are seen with CBS silencing in ovarian cancer cells [111] and with 3-MST silencing in hepatoma cells [90]. Likewise, AOAA, a pharmacological inhibitor of CBS, or HMPSNE, a pharmacological inhibitor of 3-MST, suppresses electron transport and mitochondrial bioenergetics in various cancer cell types, while supplementation of these enzymes’ respective substrates further stimulates these processes [55,56,67,70,90,111,112,113]. While H_2_S, on its own, is unable to initiate or maintain mitochondrial electron transport, it balances and enhances the effects of the physiological, glycolysis-derived electron donors such as NADH and FAD_2_, which physiologically deliver electrons to mitochondrial electron transport Complexes I and II. 

The mechanism of H_2_S-mediated mitochondrial electron donation involves the reaction of H_2_S with SQR [66,89,90,114,115,116,117]. The SQR mechanism appears to be evolutionary conserved, as various bacteria are also utilizing SQR for H_2_S “detoxification” [118,119]; as mentioned earlier, such parallels make sense due to the evolutionary bacterial origin of mitochondria. Hypoxia acts as a stimulus to induce an upregulation of SQR expression [66]—perhaps as a potential mechanism by which tumor cells attempt to maximize the bioenergetic stimulatory impact of H_2_S. However, it must be pointed out that, in a cell-type dependent manner, and especially when cells are exposed to higher [exogenous] H_2_S concentrations, the electron flow from SQR can also occur in the opposite direction (i.e., reverse electron transport) [89,120]; such a mechanism, in a cancer cell, would not be useful to support electron transport, proton pumping or ATP generation, but rather, would stimulate mitochondrial ROS generation.

In addition to direct electron transport stimulation, H_2_S can also directly increase the catalytic activity of ATP synthase, via cysteine sulfhydration [91,92]. This effect makes functional sense; increased mitochondrial electron transport would be expected to lead to a consequent increase in the proton gradient between the two sides of the mitochondrial inner membrane; this proton gradient, would, in turn, be better “harvested” if the specific activity of ATP synthase is also increased.

H_2_S has been shown to act as an inhibitor of various cAMP and cGMP phosphodiesterases; this effect is important in mediating many physiological actions of H_2_S, such as vasodilatation and angiogenesis [73,121,122,123,124,125,126]. We have demonstrated that H_2_S is an inhibitor of the mitochondrial form of cAMP phosphodiesterase (PDE2A), which, in turn, elevates mitochondrial cAMP levels and stimulates mitochondrial electron transport [93]. cAMP in mitochondria is known to activate various cAMP-dependent kinases (e.g., protein kinase A), which, in turn, phosphorylates (and consequently activates) various key proteins in the mitochondrial electron transport chain [127,128,129,130,131]. Thus, a H_2_S-induced elevation in intramitochondrial cAMP may be a further mechanism by which cancer cells may maximize their mitochondrial electron transport and ATP generation.

**Figure 1:**
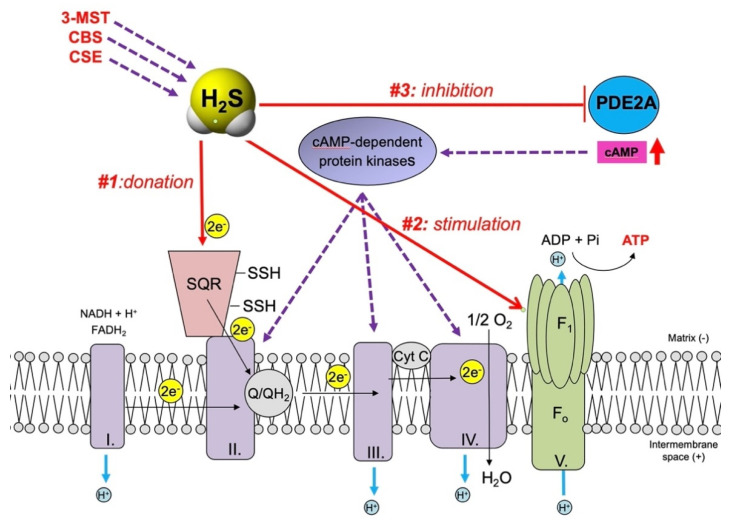
Mechanisms by which mitochondrial H_2_S (produced by CBS, CSE or 3-MST) can stimulate mitochondrial electron transport and aerobic ATP generation in cancer cells. **#1**: H_2_S acts as a direct electron donor at the level of SQR, which feeds electrons into the electron transport chain at the level of Complex II. **#2**: H_2_S inhibits intramitochondrial cAMP phosphodiesterase; this results in an elevation of intramitochondrial cAMP, which, in turn, phosphorylates electron transport chain proteins via the activation of intramitochondrial cAMP-dependent protein kinases. #**3**: H_2_S acts as direct stimulator of ATP synthase activity via sulfhydration of the α subunit (ATP5A1) at Cys 244 and Cys 294.

The above three principal mechanisms by which H_2_S can enhance mitochondrial electron transport and ATP production in cancer cells are shown in Figure 1. Additional mechanisms whereby H_2_S may contribute to the stimulation of cancer cell metabolism are summarized in Table 2. Some of these mechanisms relate to the regulation of mitochondrial organization (discussed below). H_2_S can also stimulate glycolysis (another key energetic process in cancer cells, which, however, is also intimately linked to the support of mitochondrial function, because it supplies electron donors to the mitochondria). Several sulfhydration targets (e.g., LDH-A and various sirtuins) can be activated by H_2_S, which, in turn, will lead to an increase in cellular NAD^+^ levels. 

A further, recently discovered mechanism relates to the upregulation of ACLY by H_2_S [71]. So far, this mechanism has only been investigated in the context of colon epithelial cells’ mesenchymal-epithelial transition process: the H_2_S-mediated upregulation of ACLY serves to maintain the cells in the mesenchymal state, at least in part through the upregulation of the Wnt pathway [71]. However, given the broad bioenergetic roles of ACLY, and its known upregulation in cancer cells [132,133,134], the H_2_S-ACLY axis may have further bioenergetic and metabolic implications. 

“Broader” (i.e., more generalized) mechanisms by which H_2_S can protect mitochondria may be related to its antioxidant effects, which, in part, may relate to direct reactions of H_2_S with various reactive species, and, in part, may be due to a globalized upregulation of antioxidant processes—at least in part via NRF2 and p66Shc activation [19,96,97,98,99,100]. 

## 4. H_2_S, a Regulator of Mitochondrial Dynamics in Cancer Cells

H_2_S has been shown to regulate all significant aspects of mitochondrial dynamics: mitochondrial fusion, mitochondrial fission, mitochondrial macroautophagy/mitophagy, and mitochondrial biogenesis) [87,94,95,135,136,137,138,139,140,141,142,143,144,145,146]. The majority of the published studies indicate that H_2_S (especially in lower concentrations) tends to stabilize and preserve mitochondria, and, in many cases, can also stimulate mitochondrial biogenesis. Importantly, most of the studies conducted to date utilized *exogenous* H_2_S administration (as opposed to investigating the role of endogenously produced H_2_S). Moreover, most studies focus on pathophysiological conditions other than cancer. In the current section, primarily the body of evidence that directly relates to cancer will be discussed. 

The first study, investigating the role of endogenous CBS on the organization of mitochondria in a cancer cell was conducted in ovarian cancer cells by Bhattacharyya and colleagues in a human ovarian cancer cell line (OvCa). These cells contain predominantly fused, elongated mitochondria. After siRNA-mediated silencing of CBS, the mitochondria exhibited predominantly spherical morphology, with increased individual unbranched populations and impaired mitochondrial network quality (i.e., fewer average branches per network and shorter average network branch length). These data indicate that CBS (via its enzymatic product, H_2_S) protects ovarian cancer cells against mitochondrial fragmentation; this effect may be important in maintaining mitochondrial function. The molecular mechanism that was implicated in the mitochondrial quality control in OvCa cells was the regulation of mitofusin 2 (MFN2) stability by CBS-derived H_2_S via a JNK-mediated regulation of MFN2 degradation via the via the ubiquitin-proteasome system [94]. (A similar MFN2-related mechanism has recently also been implicated in the maintenance of mitochondrial integrity in endothelial cells [146]). In an independent study, performed in N2a cells (a murine neuroblastoma cell line), H_2_S (in this case, administered exogenously to the cells) was found to inhibit mitochondrial fission; in this case the molecular mechanism was attributed to the downregulation of dynamin 1 like protein (Drp1) mRNA and protein expression by H_2_S, most likely through the modulation of ERK1/2 activity [95]. 

There are multiple H_2_S-regulated pathways that can influence mitochondrial quality control in various cell types. For instance, in murine hepatocytes, H_2_S was shown to upregulate peroxisome proliferator activated receptor-γ coactivator-related protein (PPRC) and peroxisome proliferator activated receptor gamma coactivator-1α (PGC-1α) which, in turn, stimulates mitochondrial biogenesis [141]. In the diabetic heart, ubiquitin specific peptidase 8 (USP8) has been implicated: H_2_S was found to increase the association of parkin with USP8. In turn, USP8 (a deubiquitination enzyme) was shown to promote the association of parkin to damaged mitochondria to augment mitophagy [145]. In another study focusing on cardiac myocytes, H_2_S-stimulated mitochondrial biogenesis was shown to involve AMP-activated protein kinase (AMPK) activation and subsequent induction of PGC1α signaling [87]. The various mechanisms by which H_2_S stimulates mitochondrial DNA repair and maintain mitochondrial DNA integrity (see below) can also play an indirect role in the maintenance of mitochondrial structural integrity. Future studies are needed to test whether the above-mentioned pathways are connected to each other in the regulation of mitochondrial dynamics in cancer cells.

## 5. H_2_S, A Stimulator of Mitochondrial DNA Repair in Cancer Cells

The regulation of DNA integrity is another example where the bell-shaped or biphasic effects of H_2_S are prominently featured. It has been known, for at least two decades, that exposure of high concentrations of H_2_S can induce DNA damage, while lower concentrations of H_2_S (i.e., endogenously generated H_2_S) can stimulate DNA repair. The nature of the H_2_S-induced DNA damage—predominantly characterized in the context of nuclear, rather than mitochondrial DNA—is, to a significant part, indirect, i.e., related to the intracellular generation of secondary, reactive oxygen species [147,148,149,150,151,152,153]. The molecular mechanisms of H_2_S-stimulated nuclear DNA repair are complex; multiple pathways and mechanisms (including PARP11 and g-H2AX foci formation, PCNA, CHK2, Ku70, Ku80, and DNA polymerase-d) have been implicated; this topic has been recently covered in a comprehensive review [154]. The subsequent paragraph of the current review will concentrate on the role of H_2_S in the regulation of mitochondrial DNA repair.

Perhaps the best proof for the bacterial evolutionary origin of mitochondria is the existence, structure and function of the mitochondrial DNA. Similar to bacterial DNA, mitochondrial DNA consists of a small, circular DNA structure which is not protected by histones (and which, therefore, is substantially more sensitive to oxidative damage than the nuclear DNA). The mitochondrial DNA has only approximately 16,500 base pairs, and it only encodes 13 proteins (as well as 22 tRNAs, and 2 rRNAs). The mitochondrially encoded proteins are essential protein components of the mitochondrial electron transport chain complexes. (From an evolutionary standpoint, it appears that many more mitochondrial proteins that were originally encoded on the mitochondrial DNA are now encoded by the nuclear DNA, but a select number of proteins remain mitochondrially encoded, most likely in order to maintain a rapid local control of mitochondrial function). Mutations in mitochondrial DNA disrupt the transcription of mitochondrially encoded proteins, which, in turn, can disrupt mitochondrial protein synthesis (and, consequently, mitochondrial function) in the short term and mitochondrial dynamics and organization in the long term [155,156,157]. 

The “grand total” of the literature on the role of H_2_S in the regulation of mitochondrial DNA repair consists of three published articles [99,158,159]. The first report, conducted in endothelial cells, demonstrates that AP39, a mitochondrially targeted H_2_S donor, attenuates the degree of mitochondrial DNA damage and accelerates the recovery of mitochondrial DNA integrity after oxidative damage [99]. However, this study did not investigate the underlying mechanisms of the H_2_S donor’s action. The second report, conducted in murine smooth muscle and aorta tissue focused on the role of CSE-derived H_2_S in the regulation of mitochondrial DNA copy numbers, mitochondrial content, mitochondrial-specific mRNAs (MT-CO1, CytB, and Atp 6), and implicated a role of mitochondrial transcription factor A mRNA and protein expression (TFAM) in these processes. The study concluded that H_2_S, via the regulation of DNA methyltransferase 3A (Dnmt3a) expression, and the consequent regulation of TFAM promoter methylation, is involved in the stimulation of mitochondrial DNA repair [158]. The most recent study (and, to date, the only published report focusing on the role of H_2_S in mitochondrial DNA repair in cancer cells) was conducted by our group in A549 lung adenocarcinoma cells [159]. These cells show an increased expression of all 3 H_2_S-producing enzymes. Oxidative mitochondrial DNA damage in these cells was increased and/or the efficacy of the DNA repair was impaired when the cells’ H_2_S biosynthesis was suppressed (either by treating of the cells with the pharmacological CBS inhibitor AOAA or after siRNA-mediated silencing any of the three major H_2_S-producing enzymes). The mechanism of H_2_S’ action to stimulate mitochondrial DNA repair was linked to the ability of H_2_S to induce the sulfhydration of the mitochondrial DNA repair enzyme EXOG (on Cys 76), which, in turn, promoted the assembly of a mitochondrial DNA repair complex (including EXOG, APE1 and Lig3) [159]. 

Clearly, the current body of knowledge on the role of H_2_S in regulating mitochondrial DNA integrity (or replication) or other mitochondrial DNA-related processes (e.g., the transcription or translation of mitochondrially encoded proteins) in cancer cells is minimal or non-existent: thus, future work will be needed to investigate these processes. Nevertheless, even from the current body of data, an interesting paradox emerges: on one hand, H_2_S production in cancer cells is upregulated, and mitochondrial DNA repair is activated. On the other hand, there are reports that show that in cancer cells, in fact, the mitochondrial DNA integrity is impaired [160,161]. It is conceivable that the increased DNA repair capacity of the cancer cell is unable to keep up with the extent of DNA damage (which is also increased in cancer cells, due to a multitude of processes including increased cellular ROS/RNS generation). A possible rationalization may be related to the previously mentioned “short-term thinking” of the cancer cell: by maximizing bioenergetic capacity in the short-term, cellular integrity (including DNA integrity) may be “expandable”. Possibly, survivable mutations may even be beneficial to the tumor tissue as a whole (although not necessarily to individual cancer cells), as they might produce clones that are resistant to the body’s own immunological tumor elimination processes or to chemotherapeutic agents [160,161].

## 6. Anticancer Effects of Pharmacological H_2_S Donation 

**Figure 2:**
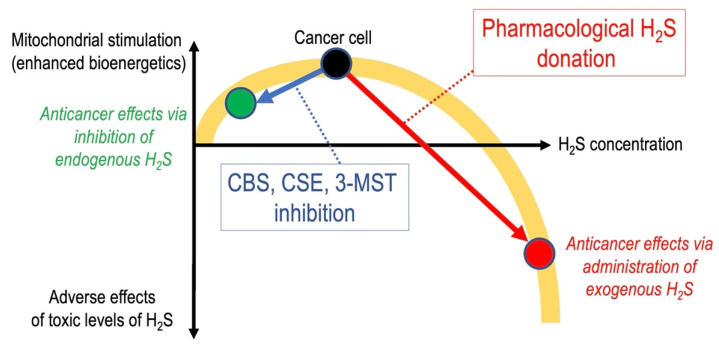
Due to the bell-shaped/biphasic pharmacological character of H_2_S, pharmacological inhibitors of H_2_S biosynthesis (via the removal of the stimulatory effects of endogenously produced, CBS, CSE or 3-MST-derived H_2_S) as well as exogenous, pharmacological H_2_S donor compounds (via stimulation of multiple cytostatic and cytotoxic mechanisms) can exert anticancer effects.

In line with the bell-shaped or biphasic effects of H_2_S, a significant body of data demonstrates that H_2_S donor compounds can exert anticancer effects by suppressing cancer cell metabolism and inducing cancer cell death. As previously discussed, [28,39,40,81,162], these cytotoxic H_2_S effects are do not invalidate the mechanisms and pathways discussed in the current article; while endogenously produced H_2_S maintains and supports a variety of beneficial (for the cancer cell, that is—i.e., not for the tumor-bearing host) processes (such as DNA repair, mitochondrial ATP generation, etc.), exogenously administered H_2_S donors reach high local concentrations, which are cytotoxic to cancer cells (but also to any other cell type) (Figure 2). The various chemical classes of anticancer H_2_S donors, their cellular actions (which, in some cases include the initiation of mitochondrial cell death pathways), and the potential difficulties with testing and developing such compounds (e.g., the theoretical and practical problems around selective targeting of the tumor cells with H_2_S donors in vivo) are extensively discussed in specialized reviews [163,164,165,166,167,168,169,170] and will not be reiterated in the current article. 

## 7. Additional Mitochondrial Roles of H_2_S in Cancer Cells

It is likely that the stimulation of mitochondrial electron transport, ATP generation, mitochondrial dynamics and mitochondrial DNA repair by endogenous H_2_S are not stand-alone processes, but, rather, they are part of a coordinated broad metabolic reprogramming process of the cancer cell. In fact, H_2_S may play an active role in this reprogramming process. For instance, H_2_S has been reported to sulfhydrate (activate) GAPDH to stimulate glycolysis [84]. An additional, H_2_S-regulated bioenergetic pathways that may become upregulated in cancer cell include the nicotinamide phosphoribosyltransferase (Nampt) (which has been implicated in the cancer cells’ ability to recover from hypoxic or oxidative damage) [54]. Moreover, metabolomic studies indicate that H_2_S can stimulate the activity of multiple Krebs cycle enzymes [56,58]. H_2_S was also found to upregulate glucose uptake in various cell types [171,172,173,174]; this may be very important to support the high glucose utilization of the cancer cell. Multiple metabolomic studies and genome wide gene expression studies [56,58,174] suggest that endogenously produced H_2_S plays a role in the reprogramming of the pyrimidine and purine metabolism, amino acid metabolism, nicotinate and nicotinamide metabolism, fatty acid metabolism, glutamate metabolism, the urea cycle, and several other pathways. The target enzymes involved in these processes remain to be characterized in the future. The available “sulfhydrome libraries” list thousands of sulfhydrated cellular proteins [44,45,46,47]. Although, for the majority of these proteins, functional follow-up studies remain to be conducted, it is important to point out that many of these sulfhydratable proteins are involved in the regulation of the above-mentioned biochemical and metabolic processes.

Endoplasmic reticulum stress (ER stress) has been implicated in the pathophysiology of many forms of cancer; this process also known to have a close and complex interrelationship with mitochondrial function/dysfunction [175,176,177]. Exogenous and endogenous H_2_S has been demonstrated to regulate ER stress [178,179,180,181]; deeper mechanistic aspects of this interrelationship remain to be investigated in the future. In addition (and, at least in part, in the context of ER stress) the potential role of cancer-cell-derived H_2_S in the regulation of mitochondrial K_ATP_ channels and mitochondrial aspects of cellular calcium handling should also be investigated in the future, given the fact that several studies implicate a regulatory role of H_2_S in these processes [182,183,184,185].

Many investigators consider CBS, CSE and 3-MST as the sole sources of H_2_S in mammalian cells. However, there are additional sources of H_2_S, the function of which remains largely unexplored in the pathophysiology of cancer. For instance, there are non-enzymatic sources of H_2_S; however, these are difficult to investigate for practical reasons (e.g., the lack of selective H_2_S scavengers). Moreover, several additional enzymes have been identified as mammalian sources of H_2_S; these include D-amino acid oxidase (DAO) in the kidney and gut [185,186], cysteinyl-tRNA synthetases (CARSs) [44], and selenium-binding protein 1 (SBP1) [187,188]. The regulation of DAO, CARSs and SBP1 in cancer and the functional role of the associated H_2_S/polysulfide production remains to be explored in future studies.

Clearly, many questions remain to be addressed in the context of the above-discussed processes. One of them relates to the mechanism(s) involved in the upregulation of H_2_S biosynthesis in cancer cells (in general, and with respect to potential translocation of H_2_S-producing enzymes into the mitochondria). CBS and 3-MST expression may be regulated both at the level of transcription, as well as at the level of degradation/protein stability [31,35]; the importance of these mechanisms in various cancers remain to be further defined. It will be also interesting to assess whether the increased H_2_S biosynthesis in cancer cells is linked to the well-known global reprogramming of substrate (cysteine, homocysteine) biosynthesis and/or cell uptake in cancer. Clearly, cancer cells undergo a global reprogramming of sulfur metabolism [48,49], and the mechanisms discussed in the current article must be placed into this broader context. 

## 8. Conclusions and Implications

In cancer cells, upregulation of various H_2_S-producing enzymes occurs in various cellular compartments (including the mitochondria), which raises intracellular (including intramitochondrial) H_2_S levels. H_2_S, in turn, stimulates mitochondrial electron transport, ATP generation, regulates mitochondrial dynamics and promotes mitochondrial DNA repair: all of these processes serve the extreme bioenergetic demand of the cancer cell. Pharmacological inhibition of H_2_S generation, which can impair the cancer cell’s mitochondrial function (and, more broadly, it can disrupt the cancer cell’s bioenergetic supply) emerges as a potential novel anticancer therapeutic concept.

## Figures and Tables

**Table 1:** Changes in H_2_S-producing enzymes in various types of cancer.

Cancer Type	Upregulation of H_2_S-Producing Enzyme(s)
Biliary tract carcinoma	CBS ↑
Bladder urothelial cell carcinoma	CSE ↑, CBS ↑, 3-MST ↑
Breast cancer	CBS ↑
Colon cancer	CSE ↑, CBS ↑↑
Gastric cancer	CSE ↑, CBS ↑
Glioma	3-MST ↑
Hepatocellular carcinoma	CSE ↑, CBS ↑
Leukemia, lymphoma	CBS ↑
Melanoma	CSE ↑, 3-MST ↑
Myeloma	CBS ↑
Ovarian cancer	CBS ↑↑
Oral squamous cell carcinoma	CSE ↑, CBS ↑, 3-MST ↑
Prostate cancer	CSE ↑, CBS ↑↑
Renal cell carcinomas	CSE ↑, CBS ↑, 3-MST ↑
Thyroid carcinoma	CSE ↑, CBS ↑↑

**Table 2:** Selected, H_2_S-activated enzymatic targets with potential relevance for cancer cell metabolism.

Target	Effect	Functional Consequence	Reference
Glyceraldehyde-3-phosphate dehydrogenase (GAPDH)	Activation via sulfhydration	Stimulation of glycolysis	[84]
Sirtuin 1, Sirtuin 3 (Sirt1, Sirt3)	Activation via sulfhydration	Elevation of cellular NAD^+^	[85,86]
Lactate dehydrogenase A (LDH-A)	Activation via sulfhydration	Elevation of cellular NAD^+^	[60]
Protein phosphatase 2A (PP2A)	Inhibition via sulfhydration	Stimulation of AMP kinase	[87]
ATP citrate lyase (ACLY)	Upregulation via promoter activation	Stimulation of acetyl-CoA synthesis	[71]
Sulfide quinone oxidoreductase (SQR)	Electron donation	Stimulation of mitochondrial electron transport	[66,88,89,90]
F0F1 ATP synthase (Complex V)	Activation via sulfhydration	Stimulation of ATP synthesis	[91,92]
Mitochondrial cAMP phosphodiesterase (PDE2A)	Inhibition via sulfhydration and dimerization	Increased mitochondrial cAMP content, stimulation of mitochondrial ATP synthesis	[93]
Mitofusin 2 (MFN2)	Upregulation through inhibition of its proteosomal degradation	Stimulation of mitochondrial biogenesis	[94]
Dynamin 1 like protein (Drp1)	Upregulation via ERK1/2	Stimulation of mitochondrial biogenesis	[95]
Reactive oxygen and reactive nitrogen species (ROS, RNS)	Neutralization of ROS/RNS via direct interactions and via upregulation of antioxidant systems through NRF2 activation and p66Shc sulfhydration	Protection against mitochondrial oxidative stress	[19,96,97,98,99,100]
DJ-1	Sulfhydration, which prevents its oxidative inactivation	Maintenance of mitochondrial redox balance	[45]

## Data Availability

Not applicable.
